# From Promise to Practice: Harnessing AI’s Power to Transform Medicine

**DOI:** 10.3390/jcm14041225

**Published:** 2025-02-13

**Authors:** Ariana Genovese, Sahar Borna, Cesar A. Gomez-Cabello, Syed Ali Haider, Srinivasagam Prabha, Maissa Trabilsy, Antonio Jorge Forte

**Affiliations:** 1 Division of Plastic Surgery, Mayo Clinic, Jacksonville, FL 32224, USA; 2 Center for Digital Health, Mayo Clinic, Rochester, MI 55905, USA

Artificial intelligence (AI) is not merely a tool for the future of clinical medicine; it is already reshaping the landscape, challenging traditional paradigms, and expanding the horizons of what is achievable in healthcare. From revolutionizing disease diagnostics [1] to streamlining patient education [2], AI-powered solutions hold the potential to enhance accuracy, efficiency, and equity in healthcare delivery. These advancements are not just tools; they serve as lifelines for a world facing increasingly complex health challenges. However, despite their promise, these innovations encounter significant obstacles that, if left unaddressed, could undermine their transformative potential.

The central challenge is evident: as AI tools become more sophisticated, our capacity to integrate them ethically, equitably, and effectively into clinical practice must evolve in tandem. This editorial explores the remarkable progress in AI-driven medicine, identifies critical gaps that hinder its full potential, and highlights the collaborative efforts necessary to build a patient-centered future.

Among the most notable advances in AI-driven medicine are its applications in diagnosis and predictive analytics, which are redefining clinical precision and decision-making. Deep convolutional neural networks (DCNNs) have achieved dermatologist-level accuracy in classifying skin lesions, making high-quality diagnostics more scalable and accessible [3]. In oncology, artificial intelligence has matched expert radiologists in diagnosing thyroid cancer via ultrasound, while also offering enhanced specificity [4]. Beyond diagnosis, machine learning models are transforming predictive analytics, providing personalized insights such as prognostic predictions for colorectal cancer that surpass the capabilities of traditional methods [4]. Additionally, large language models (LLMs) such as ChatGPT demonstrate the versatility of AI by offering valuable support in emergency medical scenarios, from generating differential diagnoses to proposing management strategies [5]. Collectively, these innovations illustrate how AI not only augments clinical practice, but also actively addresses some of the most intricate challenges in modern healthcare.

Beyond diagnostic and predictive applications, AI is driving improvements in clinical workflows, reducing administrative burdens, and enhancing patient engagement, thereby improving operational efficiency. Generative AI, including natural language processing and automatic speech recognition, can alleviate clinician workload by automating SOAP and BIRP note creation while maintaining documentation quality [6]. The AI-powered translation tool, Google Translate, has shown efficacy in patient–provider bilingual interactions, with 90% of Spanish-speaking patients able to communicate pain and nausea using neural machine translation [7]. Automated machine learning (autoML) is also emerging as a valuable asset in managing electronic health records, enhancing efficiency in data processing and clinical decision support [8]. In surgical settings, ChatGPT-4o-driven instrument recognition has demonstrated nearly 90% accuracy in broad classifications, highlighting its potential to improve workflow efficiency and procedural safety [9]. These advancements collectively support a more patient-centered healthcare system by enabling clinicians to dedicate more time to direct patient care while leveraging AI to optimize routine processes. Figure 1 expands upon the potential applications of artificial intelligence within healthcare.

However, these advancements are not without challenges. Many AI systems operate as “black boxes”, offering decisions without transparency—a major barrier to clinical trust [11]. Moreover, their real-world performance can falter due to biases in training data [12], leaving marginalized populations at risk of misdiagnosis or substandard care. For instance, algorithms trained on datasets skewed toward specific demographics may fail to generalize across diverse patient populations [13]. In parallel, digital health technologies face challenges in scalability and usability [14]. Tools that thrive in research environments may struggle in overstretched healthcare systems [14], where clinicians lack the time, resources, or training to adopt them effectively. Additionally, regulatory frameworks for AI in healthcare remain underdeveloped [15]. Unlike pharmaceuticals, which typically require rigorous trials, many AI-enabled decision support tools are approved and deployed without randomized clinical trial (RCT) evidence demonstrating improved patient outcomes [16]. This lack of robust evaluation mechanisms raises questions about safety, reliability, and accountability that must be answered to build confidence in AI-driven care. These limitations highlight an urgent need for solutions that prioritize not only technological sophistication, but also real-world feasibility.

Recognizing these gaps, this Special Issue examines the challenges and opportunities of AI in clinical medicine, exploring advancements and identifying areas for future research. By presenting diverse perspectives, it helps bridge the gap between innovation and real-world feasibility, offering insights into AI’s evolving role in clinical medicine.

As AI becomes increasingly embedded in clinical decision-making, ethical considerations must remain central to its development and deployment. One of the most pressing concerns is the shifting nature of clinical responsibility: when AI-driven tools guide treatment decisions, the lines of accountability between algorithms, clinicians, and healthcare institutions become increasingly blurred. Ensuring human oversight is crucial to prevent overreliance on automated recommendations while maintaining physician autonomy. Additionally, the use of patient data to train AI models raises concerns about privacy, informed consent, and transparency [17,18]. Without robust safeguards, these technologies risk deepening existing disparities rather than mitigating them. Addressing these ethical challenges requires a multidisciplinary approach, where ethicists, policymakers, and clinicians collaborate to create transparent regulatory frameworks that prioritize patient welfare alongside technological advancement.

To fully harness AI’s potential in healthcare, future research must not only address its current limitations, but also pave the way for responsible and effective integration. A key priority is improving AI interpretability and clinician trust. While explainable AI (XAI) methods, such as attention mechanisms and model visualization techniques, have shown promise in making AI predictions more transparent [19], further work is needed to ensure that these approaches are clinically meaningful and actionable. AI systems must provide explanations that align with medical reasoning, allowing clinicians to confidently incorporate AI-driven insights into patient care.

Another critical focus is bias mitigation. Simply diversifying training datasets is insufficient; AI models must incorporate real-time bias detection and adaptive learning mechanisms to prevent disparities from being perpetuated. Additionally, regulatory frameworks should establish mandatory fairness audits and bias assessments before AI tools are widely deployed, ensuring that AI benefits all patient populations equitably.

Beyond model development, research must explore how AI integrates into real-world clinical workflows. Many AI-driven solutions perform well in controlled environments but face challenges in clinical adoption [14]. Prospective studies should evaluate how AI influences physician decision-making, impacts clinician workload, and affects long-term patient outcomes. Understanding these factors will be essential for optimizing implementation strategies and ensuring AI enhances, rather than disrupts, existing healthcare systems.

Finally, regulatory oversight must evolve alongside AI’s growing role in medicine. Unlike traditional medical interventions, AI systems can continuously learn and adapt, creating challenges for validation and accountability. Emerging frameworks suggest real-time monitoring of AI performance post-deployment [20,21], but further research is needed to assess the feasibility and effectiveness of such approaches in clinical practice.

By addressing these critical areas, future research can drive AI innovations that improve clinical decision-making, enhance patient outcomes, and reinforce AI’s role as a trusted and equitable tool in modern healthcare.

AI and digital technology represent the most significant leap forward in clinical medicine in decades. However, their potential will remain unrealized unless we confront their limitations head-on. We stand at a crossroads: Will we allow these tools to be deployed hastily, exacerbating inequities and distrust, or will we invest the time and effort needed to ensure that they truly serve patients, clinicians, and society?

This moment demands more than optimism; it demands action. By addressing biases, prioritizing explainability, and fostering collaboration, we can unlock a future where AI not only augments clinical medicine, but transforms it. The opportunity is ours, but only if we rise to meet it.

## Figures and Tables

**Figure 1:**
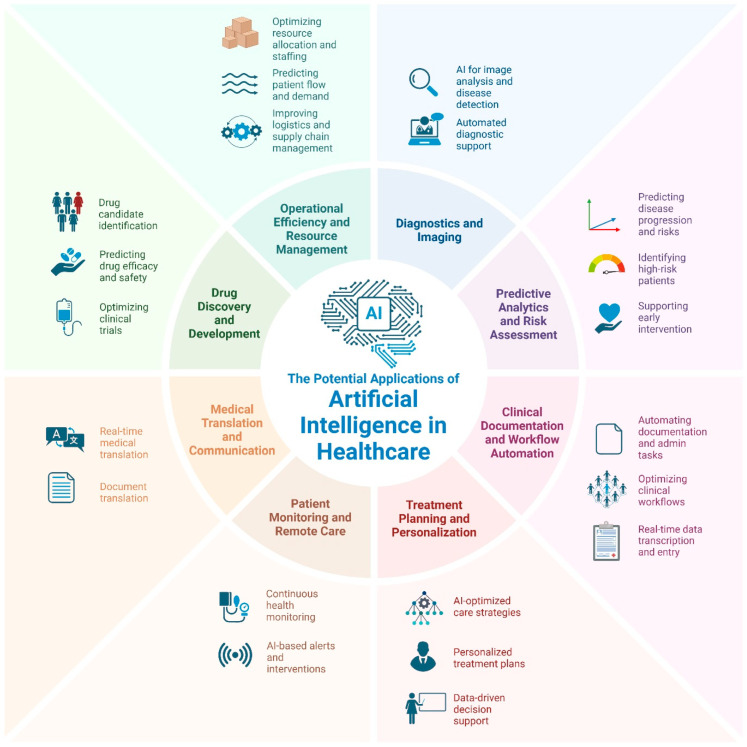
The potential applications of artificial intelligence in healthcare. Created in BioRender [10].
